# Exciton properties in zincblende InGaN-GaN quantum wells under the effects of intense laser fields

**DOI:** 10.1186/1556-276X-7-492

**Published:** 2012-08-31

**Authors:** Carlos M Duque, Miguel E Mora-Ramos, Carlos A Duque

**Affiliations:** 1 Instituto de Física, Universidad de Antioquia, Medellin, AA 1226, Colombia; 2 Facultad de Ciencias, Universidad Autónoma del Estado de Morelos, Ave. Universidad 1001, Morelos, CP 62209, Cuernavaca, Mexico

**Keywords:** Nitrides, Excitons, Intense laser field, Quantum wells

## Abstract

In this work, we study the exciton states in a zincblende InGaN/GaN quantum well using a variational technique. The system is considered under the action of intense laser fields with the incorporation of a direct current electric field as an additional external probe. The effects of these external influences as well as of the changes in the geometry of the heterostructure on the exciton binding energy are discussed in detail.

## Background

InGaN-based systems have revealed a high prospect for applications in optoelectronics. Although the hexagonal (wurtzite) allotropic form is the one most commonly considered, the zincblende (ZB) III-V nitrides are also very promising materials that have been obtained with high-quality crystal structure [1-4]. This is mostly due to the fact that the cubic symmetry avoids the presence of rather high spontaneous polarizations in the crystal, which are, in a greater extent, responsible for the presence of large built-in fields in wurtzite-based heterostructures, responsible for important reductions in the oscillator strength, and the optical recombination rates in that kind of systems [5,6]. However, the ZB structure in nitrides is provided with higher carrier mobilities, larger optical gain and lower threshold current density because of its smaller effective mass, and has mirror facets compatible with substrates such as GaAs [7-9]. In consequence, the ZB nitride-based heterostructures have drawn much attention in recent times [10-13].

The knowledge of exciton states is important for the correct understanding of some optical properties in the semiconducting low-dimensional systems. Investigations on excitons and related optical properties in ZB nitride low-dimensional systems have been mostly performed in quantum dots [14-18], but much less in quantum well (QW) heterostructures [19,20].

Research activities on the interaction of intense laser fields (ILF) with carriers in semiconductor nanostructures have revealed interesting physical phenomena. For instance, the presence of changes in the electron density of states in QWs and quantum well wires (QWWs) [21,22], the measurement of zero-resistance states in two-dimensional electron gases under microwave radiation [23], terahertz resonant absorption in QWs [24], and Floquet-Bloch states in single-walled carbon nanotubes [25], among others. A number of investigations on the effect of laser fields on low dimensional heterostructures have been published. The dressed atom approach was extended by Brandi et al. [26,27] to treat the influence of the laser field upon a semiconductor system. In the model, the interaction with the laser is taken into account through the renormalization of the semiconductor effective mass. The appearance of an unexpected transition from single to double QW potential induced by ILF was revealed in a theoretical study from Lima et al. [28]. Within the laser-dressed potential model, it is found that the formation of a double-well potential for values of the laser frequencies and intensities such that the so-called laser-dressing parameter *α*_0_ is larger than *L*/2, where *L* is the QW width. This fact is associated with the possibility of generating resonant states into the system’s channel as well as of controlling the population inversion in QW lasers operating in the optical pumping scheme.

The present work is concerned with the theoretical study of the effects of ILF on exciton states in single ZB nitride QWs of the InGaN-GaN prototype. The research is extended to include the additional influence of an applied direct (dc) electric field oriented along the growth direction of the system. The paper is organized as follows. In the ‘Theoretical framework’ subsection in the ‘Methods’ section, we describe the theoretical framework. The ‘Results and discussion’ section is dedicated to the results and discussion, and finally, our conclusions are given in the ‘Conclusions’ section.

## Methods

### Theoretical framework

Here, we are concerned with the effects of ILF on the binding energy of a heavy-hole exciton in a single In_*x*_Ga_1−*x*_N-GaN QW grown along the *z*-axis and in the presence of applied electric field. The envelope-function and parabolic-band approximations are assumed. The choice for the electric field orientation is $\vec{F}=(0,0,-F)$. The Hamiltonian for the confined exciton is then given as follows:

(1) $$\begin{array}{lll} \;Ĥ=\; & -\;\frac{\hbar^{2}}{2\;m_{e}^{*}}\nabla_{e}^{2}+V_{e}(z_{e})+e\;\vec{F}\cdot \;\vec{r_{e}}\; & \;\\ \; & -\;\frac{\hbar^{2}}{2\;m_{h}^{*}}\nabla_{h}^{2}+V_{h}(z_{h})-e\;\vec{F}\cdot \;\vec{r_{h}}-\frac{e^{2}}{\varepsilon \;|\vec{r_{e}}-\vec{r_{h}}|}\;,\; \end{array}$$

where $\vec{r_{e}}$ ( $\vec{r_{h}}$) is the electron (hole) coordinate, $m_{e}^{*}$ ( $m_{h}^{*}$) is the spherically symmetric electron (hole) effective mass, *ε*is the static dielectric constant, *e* is the absolute value of the electron charge, and *V*_*i*_ (*z*_*i*_) (*i* = *e*,*h*) are the QW confining potential for the electron and hole. The functional form of the potential in the absence of the ILF is given as follows:

(2) $$\begin{array}{ll} V_{i}(z_{i})=\left\{\begin{array}{cc} 0, & |z_{i}|\leq +L/2,\\ V_{i}, & +L/2<|z_{i}|\leq +L_{\infty }/2,\\ \infty , & |z_{i}|>+L_{\infty }/2. \end{array}\right) & \end{array}$$

The electron and hole effective masses and the static dielectric constant have been considered to have the same value (the one in In_*x*_ Ga_1−*x*_N) throughout the In_*x*_ Ga_1−*x*_N-GaN QW.

In order to find the eigenfunctions $\Psi (\vec{r_{e}},\vec{r_{h}})$ of the exciton Hamiltonian (Equation 1), it must be noticed that the total in-plane exciton momentum $\hat{\vec{P}}=({\hat{P}}_{x},{\hat{P}}_{y})$ is an exact integral of motion, and the exciton envelope wave function may be written as follows:

(3) $$\Psi (\vec{r_{e}},\vec{r_{h}})=\frac{\exp\left[\left(i/\hbar )(\vec{P}\cdot \vec{R}\right)\right]}{\sqrt{S}}\phi (\rho ,z_{e},z_{h})\;,$$

where *S* is the transverse area of the In_*x*_ Ga_1−*x*_N-GaN QW, $\vec{R}$, $\vec{\rho }$ are the in-plane center of mass and relative exciton coordinates, and $\vec{P}=(P_{x},P_{y})$ is the eigenvalue of the operator $\hat{\vec{P}}$. If $\vec{P}=0$ (ground state), then $\phi (\vec{\rho },z_{e},z_{h})$ is the eigenfunction of the Hamiltonian:

(4) $$\begin{array}{ll} Ĥ=\frac{{\hat{p}}_{\rho }^{\;\;2}}{2\;\mu }+{Ĥ}_{e}+{Ĥ}_{h}-\frac{e^{2}}{\varepsilon \;r}\;, & \; \end{array}$$

where $r=\;{\left[\rho^{2}+{\left(z_{e}-z_{h}\right)}^{2}\right]}^{\frac{1}{2}}$, ${\hat{\vec{p}}}_{\rho }=\hat{x}\;{\hat{p}}_{x}+\hat{y}\;{\hat{p}}_{y}$, $\mu =m_{e}^{*}\;m_{h}^{*}/(m_{e}^{*}+m_{h}^{*})$,

(5) $$\begin{array}{ll} {Ĥ}_{e}=\frac{{\hat{p}}_{z_{e}}^{\;\;2}}{2\;m_{e}^{*}}+V_{e}(z_{e})-e\;F\;z_{e}\;, & \; \end{array}$$

and

(6) $$\begin{array}{ll} {Ĥ}_{h}=\frac{{\hat{p}}_{z_{h}}^{\;\;2}}{2\;m_{h}^{*}}+V_{h}(z_{h})+e\;F\;z_{h}. & \; \end{array}$$

The method for the obtention of the electron and hole states is based on the work by Xia and Fan [29].

In order to consider the ILF effects (the polarization of the laser radiation is parallel to the *z*-direction), the so-called Floquet method is adopted [30,31]. According to this formalism, the second term at the right hand side in Equations 5 and 6 must be replaced by laser-dressed potential 〈*V*〉( *z*_*i*_*α*_0*i*_), where for _*α*0*i*_ is the laser-dressed parameter (from now on the ILF-parameter) defined as follows [32]:

(7) $$\begin{array}{lll} \alpha_{0i}\; & =\;(e\;A_{0})/(m_{i}^{*}\;c\;\omega )\; & \;\\ \; & =\;(I^{1/2}/\omega^{2})(e/m_{i}^{*}){\left(8\;\Pi /c\right)}^{1/2}\; \end{array}$$

In Equation 7, *I* and *ω*are, respectively, the average intensity and the frequency of the laser, *c* is the velocity of the light, and _*A*0_ is the amplitude of the vector potential associated with the incident radiation. A detailed discussion on the derivation of 〈*V*〉( *z*_*i*_*α*_0*i*_) is provided in other studies [28,33-37].

Under the laser effects, the last term of Equation 4—the one-center electron-hole Coulomb interaction—must be replaced by a two-center Coulomb interaction as follows:

(8) $$\begin{array}{lll} {\langle V\rangle }_{C}(z_{e},z_{h},\alpha_{0})=\; & -\;\frac{e^{2}}{2\;\varepsilon \;{\left[\rho^{2}+{\left(z_{\mathrm{eh}}-\alpha_{0}\right)}^{2}\right]}^{1/2}}\; & \;\\ \; & -\;\frac{e^{2}}{2\;\varepsilon \;{\left[\rho^{2}+{\left(z_{\mathrm{eh}}+\alpha_{0}\right)}^{2}\right]}^{1/2}}\;,\; \end{array}$$

where *z*_*eh*_ = *z*_*e*_− *z*_*h*_and *α*_0_ = (*eA*_0_)/(*μcω*).

The procedure adopted for the variational evaluation of the exciton wave function in the In_*x*_ Ga_1−*x*_N-GaN QW under the ILF effects is the one proposed by Fox et al. [38] and Galbraith and Duggan [39]. The functional

(9) $$E(\lambda )=\langle \phi (\rho ,z_{e},z_{h})|H|\phi (\rho ,z_{e},z_{h})\rangle$$

must be minimized with the use of the variational wave function:

(10) $$\phi (\rho ,z_{e},z_{h})=N\;f(z_{e})\;f(z_{h})\;\exp\left[-\lambda \;(|\vec{r_{1}}|+|\vec{r_{2}}|)\right]\;$$

where *λ* is the variational parameter. Besides, $\vec{r_{1}}=\vec{r}-(0,0,\alpha_{0})$ and $\vec{r_{2}}=\vec{r}+(0,0,\alpha_{0})$ with $\vec{r}=(x,y,z_{e}-z_{h})$.

The exciton binding energy is obtained from the following definition:

(11) $$E_{b}=E_{0}-E(\lambda_{\text{min}})\;,$$

where *E*_0_ is the eigenvalue of the Hamiltonian in Equation 4 without the Coulomb interaction term—the last one at the right hand side—and *λ*_min_ is the value of the variational parameter in which the energy in Equation 11 reaches its minimum.

## Results and discussion

Zincblende III-V nitride heterostructures are strained ones, given the lattice mismatch between the constituent materials. Although we are considering here a (001)-oriented In_0.2_ Ga_0.8_N-GaN QW configuration, the small indium content does not prevent from taking strain effects into account. In particular, there is a breaking of the degeneracy of heavy and light hole valence bands at the center of the two-dimensional Brillouin zone. In this work, we are including strain effects in the most simple way, that is, by incorporating the strain-induced shifts of the conduction and valence band edges in the unperturbed potential profile configuration for both electrons and holes (see, for instance, [40,41]). Data related with material properties and confining potential are taken from another work [42].

Considering the strain effects between the well and barrier materials, the electron and hole confinement potential have been obtained, respectively, by the following:

$V_{e}=Q\;(E_{g}^{b}-E_{g}^{w})-\Delta \;E_{c}$ and $V_{h}=(1-Q)\;(E_{g}^{b}-E_{g}^{w})+\Delta \;E_{v}$, where *Q* = 0.7, $\Delta \;E_{c}=2\;a_{c}^{w}\;e_{11}\;(C_{11}^{w}-C_{12}^{w})/C_{11}^{w}$, $\Delta E_{v}=2\;a_{v}^{w}\;e_{11}\;(C_{11}^{w}-C_{12}^{w})/C_{11}^{w}+b^{w}\;e_{11}\;(C_{11}^{w}+2\;C_{12}^{w})/C_{11}^{w}$, and _*e*11_ = ( *a*^*w*^− *a*^*b*^)/ *a*^*w*^. The super-index *w* and *b* refer to the well (In_*x*_ Ga_1−*x*_N) and barrier (GaN) materials.

In Table 1, the main used parameters are reported. Here, *m*_0_ is the free electron mass. The parameters of the In_*x*_ Ga_1−*x*_N material have been obtained by linear interpolation between InN and GaN.

**Table 1:** The effective mass parameters used in the calculations [42]

	***a***	***C***_**11**_	***C***_**12**_	***a***_***v***_	***a***_***c***_	***b***	***E***_***g***_	***m***_***e***_	***m***_***h***_	***ε***
InN	4.98	187	125	-0.7	-2.65	-1.2	0.7	0.1	0.835	9.7
GaN	4.50	293	159	-0.69	-6.71	-2.0	3.22	0.19	0.81	

The units are as follows: *a* in Å, *a*_*v*_, *a*_*c*_, *b*, and *E*_*g*_ in eV, and *m*_*e*_ and *m*_*h*_ in units of *m*_0_ (the free electron mass).

**Figure 1:**
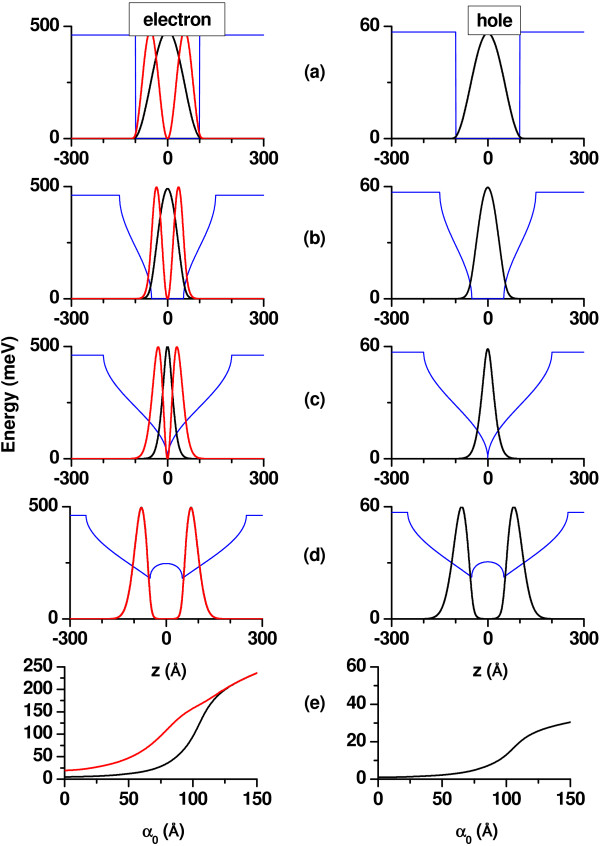
**Confinement potential and*z*-dependent amplitude of probability for the first two electron and ground hole confined states in aIn**_***0.2***_**Ga**_***0.8***_**N-GaN QW.** The results are for *L*=200 Å and have been considered several values of the ILF-parameter: *α*_0_ = 0(**a**), *α*_0_ = 50 Å (**b**), *α*_0_ = 100 Å (**c**), *α*_0_ = 150 Å (**d**). For the sake of illustration, the scale for the wave function amplitudes has been set to the same value. Graphics in row (**e**) correspond to the variation of the energies of the first two electron states (left panel) and the heavy hole ground state (right panel) as functions of the intense laser field parameter. In all cases, it is taken that *F* = 0 and *P* = 0.

The potential responsible for the confinement of electrons and heavy holes in the QW is depicted in Figure 1 for several values of the ILF parameter (Figure 1a,b,c,d). The column at the left-hand side contains the graphics that correspond to the conduction band profile, while the corresponding valence band bendings are shown in the column at the right. It is possible to observe the evolution of the QW shape associated with the change in the laser intensity—without applied dc field—by going through rows one to four in the picture. The transition from a single to a double QW potential is detected in the figures of the fourth road. We consider, of interest, to highlight that the confining potential for holes in a In_0.2_ Ga_0.8_N-GaN QW also experiences that kind of single-to-double QW transition at the value of *α*_0_reported in the current work. This is because such a feature is not present in the case, for instance, of a Ga_0.7_ Al_0.3_As-GaAs QW, in which, for the same value of the ILF parameter, the shape of the conduction band profile is very similar with that of Figure 1b [43]. Despite the greater value of the hole effective mass in the present system compared with that of the arsenide-based one, the main reason of such a difference lies in the height of the valence band confining barrier, which in the latter case is almost three times larger than the one formed in the nitride-based heterostructure studied here.

In the fifth row (Figure 1e), the evolution of the confined electron and hole levels as functions of *α*_0_ clearly show the growth in the energy values that resulted from the laser-induced deformation of the conduction and valence band potential profiles. Such modification in the QW shape involves a significant rise of the well bottom which acts by pushing up the energy levels. In the valence band, the original depth of the QW is only enough to accommodate a single heavy-hole level and, according to the basic properties of the confined one-dimensional motion, there will always be one energy level in the hole subsystem. In the conduction band, for sufficiently large laser field intensities, the first excited state is expelled from the QW, and there only remains a single confined level (the ground state one).

**Figure 2:**
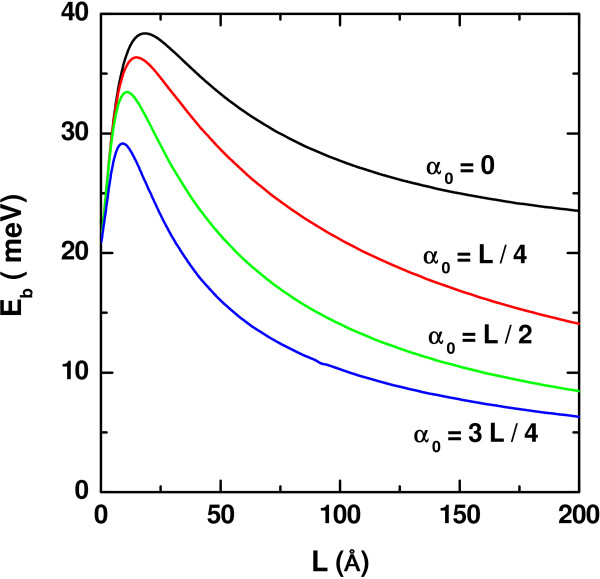
**Binding energy of heavy-hole exciton in a In**_***0.2***_**Ga**_***0.8***_**N-GaN QW.** As a function of the well-width, for several values of the ILF-parameter with *F* = 0 and *P* = 0.

Figure 2 contains our results for the heavy-hole exciton binding energy as a function of the QW width, without the application of any dc electric field and taking several values of the *α*_0_ as a parameter. The shape of the curves is typical in the case of a zincblende QW. Independent of the laser intensity, there is initially a growth in *E*_*b*_associated to the transition from a purely two-dimensional exciton to a quasi-two-dimensional one, that is, for the lower values of the well width, it favored the overlap between the confined electron and hole densities of probability, making that the expected values of the inter-carrier distance, 〈*ϕ*| *z*_*e*_− *z*_*h*_|*ϕ*〉 to be smaller, thus provoking the strengthening of the Coulombic interaction between them. As long as the QW widens, this expected value becomes larger, and the electrostatic interaction weakens, with the consequent reduction in the exciton binding energies. The decrease in *E*_*b*_for a fixed well width, *L*, observed when going from a zero laser field to a more intense one is also due to a decrease in the Coulombic correlation between both types of carriers. In fact, as can be seen from Figure 1, augmenting the laser intensity makes the allowed confined energy states to shift upwards. Therefore, the corresponding wave functions will spread over a wider interval of the coordinate, and the values of 〈*ϕ*| *z*_*e*_− *z*_*h*_|*ϕ*〉 will be larger. The kind of convergence exhibited by the curves for larger *L* reflects the increasing effect of the rigid barriers located at ± *L*_*∞*_/2 (**with***L*_*∞*_ = 600**Å**). This means that in all cases, the curves are tending toward the the exciton binding energy of an infinite barrier QW of width Ł_*∞*_, with or without a laser effect.

**Figure 3:**
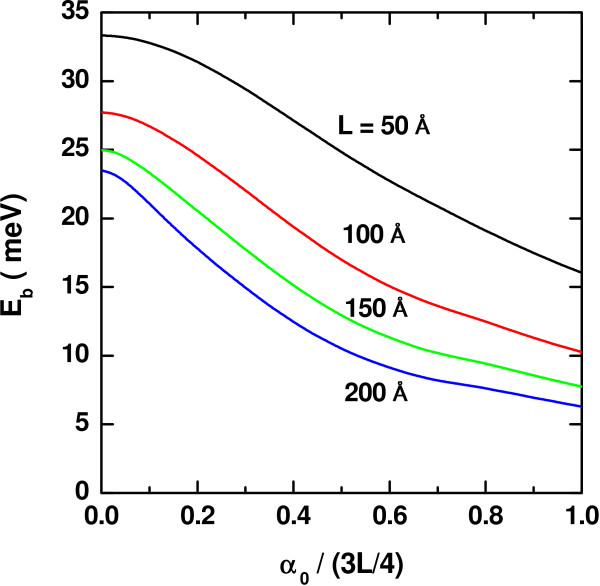
**Binding energy of heavy-hole exciton in a In**_***0.2***_**Ga**_***0.8***_**N-GaN QW.** As a function of the ILF-parameter, for several values of the quantum well-width with *F* = 0 and *P* = 0.

If an intense laser field is applied taking the QW geometry as a varying parameter, the results obtained for the heavy-hole exciton binding energy as a function of *α*_0_ are those shown in the Figure 3. They are consistent with the explanation given above regarding the weakening in the strength of the electron-hole interaction associated with the loss of confinement induced either by the increment in the laser intensity or by the enlargement of the QW size.

**Figure 4:**
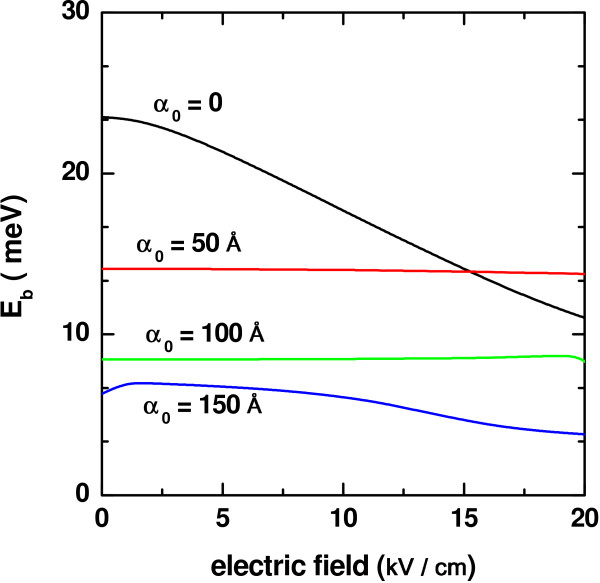
**Binding energy of heavy-hole exciton in a In**_***0.2***_**Ga**_***0.8***_**N-GaN QW.** As a function of the applied electric field with *L* = 200Å and several values of the ILF-parameter.

If a dc electric field of increasing intensity is applied to the system, keeping fixed its dimension, the heavy-hole exciton binding energy evolves as observed in the Figure 4. Once again, the value of the laser field strength appears parameterizing the different curves in the graphics. In the case of zero laser field, the variation of _*Eb*_(*F*)corresponds to an all the way decreasing function, the dc electric field amplitude. It is known that the dc field effect is mainly that of augmenting the polarization by pushing apart, spatially speaking, the carriers of opposite sign. At the same time, the rectangular QW potential profile transforms in a way that reduced the degree of carrier localization inside the well region. All this has the consequence of increasing the value of 〈*ϕ*| *z*_*e*_− *z*_*h*_|*ϕ*〉 and the corresponding fall in the Coulomb interaction. However, this particular evolution of the binding energy seem to practically disappear for the two intermediate values of the ILF parameter considered. One notices from Figure 4 that a very slight decrease in *E*_*b*_ is obtained when the value of *F* goes from zero to 20 kV/cm, if *α*_0_ is a quarter of the QW width. At the same time, what we see when *α*_0_ is equal to the half of the well width is, even, a slight increase in *E*_*b*_over almost the entire interval of *F* considered, though for the largest values of the dc field amplitude, that quantity starts showing a decreasing behavior. Hence, what is happening here is a phenomenon of compensation of the progressive augmenting of the electron-hole expected distance via the deformation of the QW potential profile obtained when combining the effects of the two kinds of externally applied fields, that is, if the effect of the dc field is to push the electronic wave function towards the left-hand side of the QW, given that the height of the barrier for electrons is significantly bigger than the one corresponding to the valence band, the displacement of the electron wave function is counteracted by the barrier repulsion (one must keep in mind that the dc field strength values considered here are not very high). On the other hand, the electric field will induce a displacement of the heavy hole towards the right. However, the QW barrier height is so small here that, thanks to the ILF-induced pushing-up effect of the energy level position, the hole density of probability can penetrate further to the left, with the consequent increment in the overlap between electron and hole wave functions. As a result of this, the expected electron-hole distance diminishes. This is the cause of the compensating effect and the apparent insensitivity of *E*_*b*_with respect to *F* for such modified QW shapes associated to such particular values of *α*_0_. Once the laser field intensity is sufficiently high (lower curve in Figure 4), the heavy-hole exciton binding energy recovers its decreasing variation as a function of the dc field strength (again due to the fall in the carrier localization), with the exception of a very slight increment noticed for very small values of *F*. Here, the combination of the slow linear change of |*eFz*|with the ILF-induced double QW shape of the confining potential (Figure 1d) leads to the kind of compensating effect mentioned above. In this case, it leads to a small reduction in 〈*ϕ*| *z*_*e*_− *z*_*h*_|*ϕ*〉 and the observed little increase in *E*_*b*_in that region.

**Figure 5:**
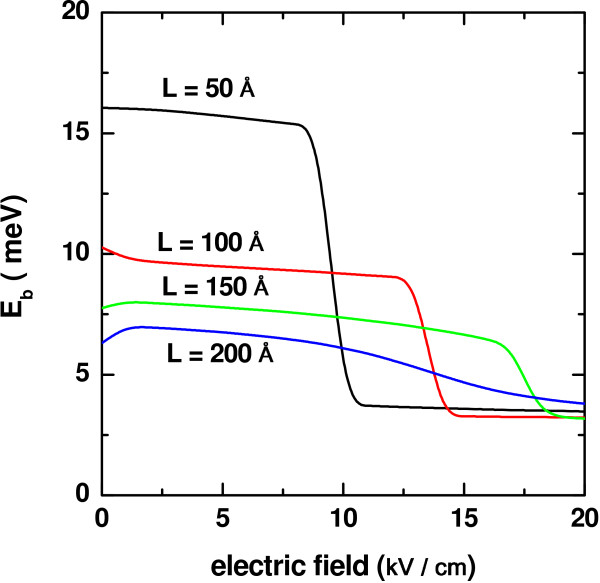
**Binding energy of heavy-hole exciton in a In**_***0.2***_**Ga**_***0.8***_**N-GaN QW.** As a function of the applied electric field for several values of the quantum well-width (*L*) with *α*_0_ = 3*L*/4.

Finally, Figure 5 shows the variation of the heavy-hole exciton binding energy as a consequence of the increment in the intensity of the *z*-oriented applied dc electric field. In this situation, the width of the QW appears and is considered as the parameter that differentiates between the curves depicted.

The configuration chosen includes an applied laser field with intensity given, in each case, by the parameter *α*_0_ = 3*L*/4. It is seen that for the two lowest values of the well width, *E*_*b*_is a slight decreasing function of *F* until a certain critical value, *F*_*c*_, of the dc field strength at which initiates an abrupt fall that leads to a constant, limit value, that remains for the rest of the increasing range of the amplitude *F*. The decrease occurring while *F* < *F*_*c*_ is justified along the same arguments expressed above with regard to the progressive enlargement of the inter-carrier average distance that associates with the loss in electron and hole confinement. The abrupt descent in *E*_*b*_has to do with the escape of one (electron or hole) of the wave functions away from the QW region, towards the infinite barrier on the side it was pushed to by the electric field. The value of the expected electron-hole distance then suffers a sudden rise which reflects in the drop of *E*_*b*_ observed. Augmenting further the dc field strength will function to cause the same effect on the other wave function in such a way that the increase in *F* will not have any other influence on the polarization because the carriers will remain confined by the infinite barriers at ± *L*_*∞*_/2. Therefore, one may see that *E*_*b*_adopts a constant value when *F* becomes large enough.

It is worth mentioning that, for all the values of *L* taken into account, setting *α*_0_ = 3*L*/4implies a great modification of the confining potential profile which, as one of the main features, presents a significantly reduced effective well depth. At the same time, the effect of confinement reduction on the carrier wave functions is more pronounced for narrower QWs, for the allowed energy levels are, initially, placed at higher energy positions. Thus, the application of the not so intense dc fields readily leads to the mentioned wave function escape. This explains why the phenomenon of abrupt change in *E*_*b*_ is manifested for smaller dc field intensities.

The curves that correspond to the two highest values of *L* in Figure 5 show an increasing behavior for the smallest electric field amplitudes. This fact relates with the reduction in 〈*ϕ*| *z*_*e*_− *z*_*h*_|*ϕ*〉 obtained as a result of the combination of the laser and dc fields on the confinement of the carriers. A small *F* associates with a slight linear deformation of the already modified (by the laser effect) potential profile. The electron and hole densities of probability are pushed in opposite directions, but the potential well barriers, not so deformed, repel them away. This has the consequence of bringing the two particles a little bit closer and, therefore, of augmenting the strength of their Coulombic interaction. However, when the dc field is augmented, the dominant influence is that causing the spatial spreading of the carrier wave functions, which leads to the decrease in *E*_*b*_. Notice that the pronounced fall is also present when *L* = 150Å, but for *L* = 200Å *E*_*b*_ is a rather smooth monotonically decreasing function of *F*, without any abrupt change. This is because the QW width is large enough to avoid the sudden escape of the wave functions and also because the limiting infinite barriers are much closer to the inner well ones.

## Conclusions

The properties of heavy-hole excitons in InGaN-GaN-based quantum wells under intense laser and applied dc electric fields are studied for a set of different values of the fields intensities and the well spatial dimensions. In general, for a fixed geometry of the unperturbed system, the exciton binding energy is a decreasing function of the intense laser field parameter and of the dc electric field, although certain combinations of the two applied field intensities may lead to a rather insensitive behavior of the binding energy with respect to the application of a dc field. It is shown that the changes of the degree of carrier confinement and of the carrier polarization associated to the influence of the laser and the dc fields are mainly responsible for the exciton properties mentioned. To our knowledge, there seem to be no previous reports on exciton properties in zincblende nitride QW induced by intense laser fields. Thus, the results of the present work might be considered as a first approximation to the subject in this kind of systems.

## Competing interests

The authors declare that they have no competing interests.

## Author’s contributions

CMD carried out the numerical work. MEMR carried out the discussion of results and writing. CAD carried out the numerical work. All authors read and approved the final manuscript.
